# Profile of Neonatal Septicaemia at a District-level Sick Newborn Care Unit

**DOI:** 10.3329/jhpn.v30i1.11274

**Published:** 2012-03

**Authors:** Rajlakshmi Viswanathan, Arun K. Singh, Chiranjib Ghosh, Sudipta Dasgupta, Suchandra Mukherjee, Sulagna Basu

**Affiliations:** ^1^Department of Neonatology, Institute of Post Graduate Medical Education and Research and Seth Sukhlal Karnani Memorial Hospital, Kolkata, West Bengal, India; ^2^Sick Newborn Care Unit, Suri Sadar Hospital, Suri, Birbhum District, West Bengal, India; ^3^Division of Bacteriology, National Institute of Cholera and Enteric Diseases, Kolkata, West Bengal, India

**Keywords:** Antibiotic resistance, Blood culture, Intensive care, Neonatal, Sepsis, India

## Abstract

Although sepsis is a major cause of morbidity and mortality among newborns in resource-poor countries, little data are available from rural areas on culture-proven sepsis. The aim of the present study was to provide information in this regard. The study reports results on the incidence and aetiology of neonatal sepsis cases admitted to a facility in a rural area in eastern India. Blood culture was done for all babies, with suspected clinical sepsis, who were admitted to the sick newborn care unit at Suri where the study was conducted during March 2009–August 2010. A standard form was used for collecting clinical and demographic data. In total, 216 neonatal blood culture samples were processed, of which 100 (46.3%) grew potential pathogens. Gram-negative infection was predominant (58/100 cases) mainly caused by enteric Gram-negative bacteria. *Klebsiella pneumoniae* was the most common Gram-negative isolate. The emergence of fungal infection was observed, with 40% of the infection caused by yeast. Gram-negative organisms exhibited 100% resistance to ampicillin, cefotaxime, and gentamicin. Amikacin and co-trimoxazole showed 95% (n=57) resistance, and ciprofloxacin showed 83.3% (n=50) resistance among the Gram-negative bacteria. Carbapenem showed emerging resistance (n=4; 6.6%). Results of analysis of risk factors showed an extremely significant association between gestation and sepsis and gender and sepsis. Gastrointestinal symptoms were highly specific for fungal infections. One-third of babies (n=29), who developed culture-positive sepsis, died. Blood culture is an investigation which is frequently unavailable in rural India. As a result, empirical antibiotic therapy is commonly used. The present study attempted to provide data for evidence-based antibiotic therapy given to sick newborns in such rural units. The results suggest that there is a high rate of antibiotic resistance in rural India. Urgent steps need to be taken to combat this resistance.

## INTRODUCTION

Globally, neonatal mortality accounts for more than one-third of deaths of children aged less than five years (1). Infection accounts for one-fourth of total neonatal deaths. About 99% of these neonatal deaths take place in low and middle-income countries (2). Bacterial infection is a significant cause of neonatal and early childhood admissions to hospitals and probably of morbidity in the community but its burden is unclear (3). Identification and treatment of newborns with infection are weak in many developing countries (4). Detailed studies on the aetiology and antibiotic resistance profile of neonatal septicaemia in rural India are uncommon. Good laboratory facilities, especially blood culture, are frequently unavailable in the rural healthcare setting, resulting in the non-availability of relevant data on culture-proven neonatal sepsis.

The present study reports results on the incidence and aetiology of neonatal septicaemia from a remote rural sick newborn care unit located in eastern India. To the best of our knowledge, this is the first such report from India.

## MATERIALS AND METHODS

The study was carried out during March 2009–August 2010 at a 20-bed level II sick newborn care unit (SNCU), established in 2005, at a district hospital of Birbhum, West Bengal, 220 km from Kolkata. About 6,000 deliveries are conducted per year at the hospital which admits both inborn and outborn babies who account for approximately 70-75% and 25-30% of admissions respectively. The unit was developed along the lines of the ‘Purulia Model’, which aims to provide facility-based sick newborn care at district hospitals, with the objective of reducing the neonatal mortality rates (5). The unit provides level II care, including oxygen by hood, resuscitation with bag and mask ventilation, phototherapy, intravenous therapy, and naso/oro-gastric feeding. Services, such as ventilation, insertion of central vascular catheters, total parenteral nutrition, or post-operative care, are not available at this unit. Neonatal mortality stands at 60 per 1,000 livebirths among inborn babies (hospital data, 2009).

The microbiology laboratory support was provided at a level III unit in a tertiary-care centre in Kolkata, which already had some experience in neonatal microbiology (6,7).

### Clinical methods

Blood culture was done for all neonates suspected to have septicaemia, whose parents gave their informed consent (Fig.). One or more of the following clinical features were considered an indication for drawing blood sample for culture (8): lethargy, apnea, tachypnea, tachycardia, hypotension, instability of temperature, poor feeding, poor perfusion, and abdominal distension. Lack of suitable laboratory facility at the site meant that septic screen could not be done. Hence, clinical criteria or presence of one or more of the following perinatal risk factors—maternal fever, prolonged rupture of the membranes for more than 24 hours, foul-smelling or meconium-stained liquor, or frequent (>3) unclean vaginal examinations, and/or having severe prematurity, or birth asphyxia necessitating active resuscitation—were taken as an indication for blood culture. Early and late onset of sepsis was defined as infection within the first 72 hours of life and >72 hours of life respectively (8). Relevant clinical data were collected in a standard format (Appendix).

**Fig.**
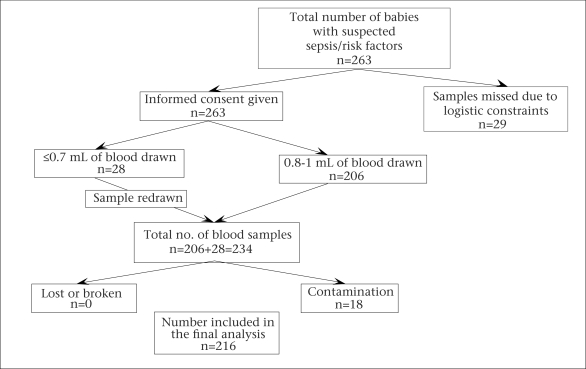
Logistics of samples collected and analyzed

### Microbiological methods

Samples were collected at the district unit and were transported to the nodal laboratory, within 4-6 hours, for further processing, that is incubation, identification, and antibiotic sensitivity testing; 0.8-1 mL of blood for culture was drawn with aseptic precautions from a peripheral vein. The inoculated vials were stored at room temperature before being transported. Blood culture was performed using the BACTEC 9050 system (Becton Dickinson, Sparks, Maryland, USA). For any culture which flagged positive, Gram-staining was performed, and subculture was done on appropriate media based on the Gram-stain: MacConkey agar w/o crystal violet and 5% sheep blood agar (Biomerieux, la balme les Grottes, France) for Gram-negative and positive organisms and 5% sheep blood agar and Sabouraud's dextrose agar for yeast isolates. Aerobic spore bearers, *Micrococcus* sp., and diptheroid bacilli were regarded as contaminants. Coagulase-negative *Staphylococcus* was considered a pathogen only when it was isolated in two blood cultures from the same patient. All the other isolates were included in the analysis. Bottles were incubated in the system for up to seven days at the end of which all the negative bottles were subcultured once on blood agar before discarding.

Identification was done by conventional methods and confirmed for Gram-negative organisms and yeasts by the mini API analyzer using ID32E kit for Enterobacteriaceae, ID32GN for non-fermenters, and ID32C for yeast isolates (bioMérieux, Marcy l’Etoile, France). The yeast isolates were sent to the Division of Medical Mycology, WHO Collaborating Centre for Identification of Fungal Isolates, ICMR Centre for Excellence, Department of Medical Microbiology, Postgraduate Institute of Medical Education and Research, Chandigarh, for molecular confirmation of identification.

The Kirby-Bauer disc-diffusion method was used for antimicrobial susceptibility testing. The guidelines of the Clinical and Laboratory Standards Institute were used for interpretation as resistant, intermediate sensitive, and sensitive (9).

Positive results were immediately informed by telephone to the district unit. Subsequently, the reports were sent by electronic mail. The hard copies of the reports were handed over on the next trip to the transport personnel.

### Analysis of data

Statistical analysis was done using the Microsoft Excel 2003 and GraphPad Prism5 software.

### Ethical approval

The Institutional Ethics Committee approved the study (memo no. 317 dated 31.3.2008).

## RESULTS

### Clinical results

The study reports the results for the March 2009–August 2010 period. Demographic data were available for 216 babies, and data regarding clinical presentation were available for 150 babies. An analysis of risk factors was carried out for the common demographic variables, such as gestational age, birthweight, sex, mode of delivery, and place of delivery―inborn or outborn in the cases of probable sepsis and culture-proven sepsis. Fisher's exact test with two-tailed p value showed an extremely significant association between gestation and sepsis and gender and sepsis (Table 1).

Symptoms, such as poor feeding and lethargy, had the sensitivity of more than 85% but their specificity and positive predictive values were poor for sepsis. Most other symptoms did not have good sensitivity or specificity, except gastrointestinal symptoms which were highly specific for fungal infections (Table 2).

Data on perinatal risk factors were available for 153 babies. Seventy babies had risk factors for sepsis, and 83 did not. Of the 70 babies with risk factors, 31 developed culture-proven sepsis, 19 of which were early in onset. Blood culture of 39 babies with risk factors did not grow any microorganism. Of the 83 babies who had no perinatal risk factors, 30 developed culture-proven sepsis, of which 22 were early in onset.

Twenty-nine (13.4%) of the 216 babies died. About 30% of the babies who developed culture-positive sepsis also expired.

### Microbiological results

During the study period, 216 neonatal blood culture samples were processed. One hundred (46.3%) blood cultures done had positive growths. Gram-negative infection was predominant (58/100 cases), mainly caused by enteric Gram-negative bacteria. Cases of infection with non-fermenting Gram-negative bacilli were few in number. The emerging fungal infection was observed, with 40% (n=40) of the infection caused by yeast (Table 3). All, except one yeast infection, occurred during an outbreak period lasting for eight months (August 2009–March 2010).

The data so far showed alarming degree of resistance to all the antibiotics commonly used for neonatal sepsis. Gram-negative organisms exhibited high degree of resistance to the World Health Organization-recommended first and second-line antibiotics (10). A 100% resistance (60/60) was noted for ampicillin, cefotaxime, and gentamicin. Amikacin showed 95% resistance (57/60) and ciprofloxacin showed 83.3% resistance (50/60) among the Gram-negative bacteria. Co-trimoxazole, which is often administered orally in the community setting (11) for neonatal sepsis, also exhibited almost full resistance (95%; 57/60) (Table 4). All the yeast isolates were sensitive to fluconazole and amphotericin B.

**Table 1.** Demographic characteristics of study subjects

Variable	Suspected sepsis	Blood culture		p value
		Negative	Positive	
Gestation (weeks)				
<37	101	24	77	<0.0001
≥37	115	92	23	
Sex				
Male	144	48	96	<0.0001
Female	71	67	4	
Birthweight (g)	
≤2,499	146	73	73	0.1448
≥2,500	70	43	27	
Mode of delivery	
Vaginal	187	99	88	0.6898
Caesarean	29	17	12	
Place of delivery				
Inborn	121	70	51	0.1728
Outborn	95	46	49	

**Table 2.** Sensitivity, specificity, positive and negative predictive values of common presenting features of sepsis

Presentation	Sensitivity	Specificity	PPV	NPV
Poor feeding	86.3	11.69	0.481	0.474
Alteration of cry	34.25	75.32	0.568	0.547
Respiratory symptoms	35.6	75.3	0.578	0.552
Gastrointestinal symptoms*	13.15	98.7	0.909	0.535
Seizures	30.13	72.7	0.512	0.523
Lethargy	85.33	14.1	0.489	0.579

*Only for yeast;

NPV=Negative predictable value;

PPV=Positive predictive value

**Table 3.** Profile of microorganisms isolated

Organism	No.
*Klebsiella pneumoniae*	32
*Escherichia coli*	11
*Enterobacter* sp.	7
NFGNB*	6
Gram-positive cocci	2
Yeast	40 (1 *Candida* and 39 *Pichia fabiannii*)
Polymicrobial infection	4 (*E. coli*–2 and *K. pneumoniae*–2)
Total	102

*Includes *Acinetobacter* sp., *Pseudomonas* sp., *Stenotrophomonas maltophilia*, and *Burkholderia cepacia* complex;

NFGNB=Non-fermenting Gram-negative bacilli

## DISCUSSION

Blood culture is the most important investigation for the management of sepsis. Due to lack of resources, it is often an irregularly-used investigation in India (12) and is largely restricted to the urban and semi-urban tertiary centres. This is particularly true in the case of neonatal sepsis. The nodal centre has since 2005 been responsible for setting up and monitoring of level II SNCUs in seven district hospitals of the state of West Bengal, India (5,13-15). In the absence of laboratory support, treatment of neonatal sepsis in these units was entirely empirical. The present study was the first step taken to provide evidence-based antibiotic therapy to newborns in such units.

**Table 4.** Antibiotic resistance profile of Gram-negative bacteria

Antibiotic	Resistance	
	No.	%
Ampicillin	60	100
Gentamicin	60	100
Amikacin	57	95
Cefotaxime	60	100
Ciprofloxacin	50	83.3
Co-trimoxazole	57	95

The high rate (46.3%) of positive blood culture in the present study was similar to that of a recent study in Tanzania where positive blood culture was found in 47% and 51.4% of neonates with early and late neonatal sepsis respectively (16).

As is seen across neonatal units in urban India (7), the most common organism isolated in the present study was *Klebsiella pneumoniae*. Non-fermenting Gram-negative bacilli which are recently emerging in neonatal intensive care units (17), were few in number. One reason for this could be: the unit studied is of level II, and interventions, such as insertion of central vascular catheters and mechanical ventilation, are not performed in this unit. This study was characterized by a virtual absence of Gram-positive infection, including Group B *Streptococcus* and coagulase-negative *Staphylococcus* (CONS). Group B *Streptococcus* is rarely documented in India (18). Infection with CONS is usually associated with the use of interventions, such as central vascular catheters, which was not prevalent in the unit studied.

Of note is the emergence of yeast infection in a unit without ventilation and central vascular access facilities. The fungal infections were part of an outbreak during August 2009–March 2010. In view of the persistence of fungal infection, a point-prevalence study was undertaken in November 2009. Specimens were collected from healthcare workers, environment, and babies admitted to the unit. We were, however, unable to pinpoint the source of infection. Standard precautions, especially hand hygiene, were reinforced. An additional step of hand hygiene, with chlorhexidine-alcohol rub, was introduced. All these perhaps contributed to the control of infection. Logistic constraints prevented tracing of further sources.

The profile of organisms causing early and late onset of sepsis was similar in the present study. This may indicate that perhaps, along with vertical transmission, the horizontal spread of infection also has a role to play in the early onset of sepsis in hospitalized neonates (7,19). The proportion of culture-positive sepsis in inborn (48.1%) and outborn (51.6%) babies was similar.

As is well-documented (20), the clinical symptoms were not specific for sepsis, barring gastrointestinal symptoms, which showed high specificity for fungal infection (21). However, the authors acknowledge that this interpretation may be of limited value since clinical data were not available for all the study babies.

The results of this study suggest that there is a high rate of antibiotic resistance even in rural India. Almost no options were left for the treatment of bacterial sepsis. When this study was initiated, cefotaxime and amikacin were being used as the empirical first-line antibiotics in the unit. This could possibly be one of the reasons for the high rates of resistance observed.

Other factors could be the easy availability and rampant use of broad-spectrum antibiotics in the presumptive treatment of infections even in rural India. It is documented that there is a strong tendency, at least in India, to start antibiotics without doing blood cultures first (22). However, it is also true that the blood-culture facility is not often available, except for the urban tertiary centres or expensive private set-ups. In the absence of case-specific information, clinicians learn to depend on empirical antibiotic regimens. Also, without a reliable blood-culture facility, physicians often err on the side of caution, preferring to treating cases which may or may not be sepsis, with antibiotics. This may lead to ‘over-diagnosis’ of sepsis and the high rate of use of antibiotics.

Results of a study in 1977 showed that, for every newborn with proven sepsis, 11-23 non-infected newborns were treated with antibiotics (23). The fact that all the cases of sepsis have not positive blood culture is probably the main reason for such usage of antibiotics. However, it cannot be denied that the clinical features of sepsis often overlap with other neonatal conditions. Unfortunately, no single diagnostic test is available to adequately differentiate sepsis from other similar conditions.

The above issues call for an urgent evaluation and audit of antibiotic use in the neonatal facilities. The infection-prevention guidelines need to be implemented at all levels of healthcare. Overcrowding and poor staffing patterns are practical problems in hospitals, such as the one where the present study was carried out. Attempts should be made to reduce this problem. It is well-documented that families in developing countries often find advice for hospitalization, which is difficult to comply with because of unpleasant past experiences of the family (24,25). One of the reasons cited is overcrowding in hospital beds (26). The danger is that, if healthcare facilities become ‘hotbeds of infection’, mothers and newborns will be further discouraged from seeking institutional care.

### Conclusions

Data on culture-positive neonatal sepsis are minimal from rural centres in India. The present study attempted to provide information in this regard. Enteric Gram-negative bacteria are the most common cause of neonatal sepsis in this set-up. An alarmingly high degree of antibiotic resistance observed calls for an urgent evaluation and development of antibiotic policy and protocols for neonatal sepsis.

## ACKNOWLEDGEMENTS

The study was supported through a grant received from the WOS (B) Scheme of the Department of Science and Technology, Government of India. Raj-lakshmi Viswanathan is the recipient of a fellowship under the WOS (B) Scheme of the Department of Science and Technology, Ministry of Science and Technology, Government of India. The authors gratefully acknowledge Mr. Deepak Singh for technical help.

## Appendix. Neonatal sepsis surveillance

District SNCU___________________/IPGMER, Kolkata

(Please tick or circle where appropriate)

- Demographic details
  **Table:**
  Hospital registration no.	Address
  Mother's name/age		
  Father's name/age	Contact no.
- Delivery details
  **Table:**
  Date of delivery	Inborn/outborn	Home/institutional delivery
  	Mode of	Home-skilled/unskilled attendant
  Gestation in weeks	delivery (normal/CS/forceps/vacuum)	Foster care―yes/no
  Birthweight/sex		Singleton/twin/triplet	Indigenous medicine―yes/no
- Risk factors
  **Table:**
  Prolonged rupture of membranes―yes/no	Premature rupture of membranes―yes/no
  Meconium-stained liquor―yes/no	Maternal fever―yes/no
  Cry spontaneous/delayed	Resuscitation required―yes/no
  Foul-smelling liquor―yes/no	Labour spontaneous/induced
  Antenatal care―yes/no	Any significant maternal health problem
- Admission details
  **Table:**
  Date of admission	Primary cause of admission
  Presenting features	
  Poor feeding	Alteration of cry
  Temperature instability	Apnea
  Tachypnea (RR >60/minute)	Grunting
  Chest retraction	Abdominal distension
  Vomiting	Hypoglycaemia
  Diarrhoea	Skin pustules
  Pus draining from umbilicus	Bleeding
  Jaundice	Lethargy
  Shock	Hypotonia/hypertonia
  Fontanelle (normal/depressed/bulging)	Abnormal movements (including seizures)
  Major congenital malformation―yes/no	Any other, please specify
- Management
  **Table:**
  IV cannula―yes/no	Blood/blood product transfusion―yes/no
  Date of blood culture sampling	Result
